# A standardized naturalistic audio stimulus dataset with unsupervised labeling

**DOI:** 10.1038/s41597-026-08034-0

**Published:** 2026-08-05

**Authors:** Anas Al-Naji, Ricarda I. Schubotz, Anoushiravan Zahedi

**Affiliations:** 1 Institute of Psychology, University of Muenster, Fliednerstr. 21, 48149 Muenster, Germany; 2 Otto Creutzfeldt-Center for Cognitive and Behavioral Neuroscience, University of Muenster, Fliednerstr. 21, 48149 Muenster, Germany

## Abstract

This study presents a standardized naturalistic audio stimulus dataset designed for use in trial-wise cognitive neuroscience, neuroimaging, and behavioral research. The dataset provides short, recognizable auditory stimuli that are normed for emotional valence and startlingness. To create such a dataset, the current study collected 291 audio files from a range of sources and standardized them to a duration of 1.5 s. A final sample of 361 participants rated the audio clips on emotional valence, startlingness, and recognizability, and subsequently freely described the audios by typing what they believed the sound to be. The text responses of the participants were embedded and clustered using an unsupervised machine-learning algorithm to derive a participant-grounded organization of auditory object categories. The results indicate that the audio clips were generally recognizable, while emotional valence and startlingness ratings varied across stimuli.

## Background & Summary

Naturalistic auditory stimuli are widely used in experimental psychology and cognitive neuroscience to study perception, affect, attention, memory, and semantic processing under conditions that approximate everyday listening^1–4^. At the same time, trial-wise behavioral and neuroimaging experiments often require stimulus materials that are short, consistently formatted, recognizable, and accompanied by sound-level annotations or ratings. These requirements allow researchers to present many trials within a fixed session, match stimuli across experimental conditions, and select subsets based on dimensions relevant to a particular study. Therefore, this Data Descriptor presents a standardized naturalistic audio stimulus dataset with participant ratings and semantic annotations. The dataset was created to provide a documented set of short auditory stimuli, psychological ratings, source information, raw participant responses, processed analysis outputs, and code for reproducing the main analyses. The stimuli were standardized to a duration of 1.5 s, and each sound was rated by participants on emotional valence, startlingness, and recognizability.

The definition of auditory objects remains debated in research. Early definitions described auditory objects as coherent constructs that emerge when the brain integrates acoustic features across time and frequency (i.e., timbre, pitch, and loudness), analogous to vision, where edges and contours can be combined to form visual objects^5^. The current consensus, however, is that auditory objects represent the perceptual units over which the auditory system organizes the acoustic world^6^. Bizley and Cohen^6^ emphasize the hierarchical nature of auditory object definition, where low-level features combine into objects, which themselves can be organized into increasingly abstract categories, and the categories can, in turn, be nested inside more general categories. For example, a dog’s bark or a bird’s call falls under broader categories such as mammals or birds, which together belong to the general category of living things. Because auditory objects and categories are important for describing how listeners organize sounds, standardized data-driven datasets that capture these distinctions can support auditory cognition research.

The dataset is situated within a broader landscape of auditory stimulus resources used in experimental, affective, and computational research. For example, the International Affective Digitized Sounds (IADS) and its expanded version (IADS-E) provide normed auditory stimuli for investigating affective responses to sound^7,8^. Similarly, the Norms for Environmental Sound Stimuli (NESSTI) provides psychological norms, including cognitive and affective ratings, for 110 environmental sounds from living and man-made sources^9^. In parallel, environmental sound datasets such as ESC-50, UrbanSound8K, AudioSet, and FSD50K have contributed substantially to research on auditory recognition, categorization, and computational sound classification^10–13^. Furthermore, validated vocal and speech datasets, such as the Montreal Affective Voices (MAV) and the Ryerson Audio-Visual Database of Emotional Speech and Song (RAVDESS), have provided standardized materials for research on nonverbal vocal affect, emotional speech, and emotional song^14,15^. The present dataset is intended as a complementary resource that documents a set of brief naturalistic auditory stimuli together with recognizability, valence, startlingness, and participant-derived semantic annotations.

To construct the dataset, 291 audio stimuli were collected from a range of sources and standardized to a uniform duration of 1.5 s. These audio clips were rated by participants on emotional valence, startlingness, and recognizability, and subsequently freely described by typing what they believed the sound to be. These responses were embedded and clustered using an unsupervised machine-learning algorithm to derive a participant-grounded organization of auditory object categories. This dataset is designed for cognitive neuroscience and neuroimaging research, as well as in the broader field of behavioral sciences.

## Methods

### Stimuli curation

Stimuli were obtained from two repositories: BBC Sound Effects^16^ and Freesound.org^17^. The BBC Sound Effects library provides sounds licensed for non-commercial use, whereas Freesound.org distributes audio under various Creative Commons licenses, including CC0 (unrestricted use), Attribution (credit required), and Attribution–Noncommercial (credit required, non-commercial use only). A complete list of credited Freesound.org contributors is provided in the supplementary materials.

The sounds were selected to adhere to predefined criteria: each sound had to have a duration of less than 1.5 s, or be amendable to trimming of 1.5 s without loss of its characteristics. Sounds that were artificial, contained high levels of static noise (e.g., audible background white noise), were discontinuous (e.g., footsteps), or were alarming (e.g., an emergency siren) were excluded from the final dataset. The final stimuli set contained 291 sounds.

The 1.5-s duration was selected as a compromise between perceptual decision demands and experimental efficiency. Because the dataset is intended primarily for cognitive neuroscience and neuroimaging paradigms, the stimuli needed to be short enough to allow many repeated trials within a fixed experimental session, while still providing sufficient acoustic information to support source-related perceptual decisions. This duration is consistent with prior work on environmental sound processing, which shows that auditory object identification - recognizing the source or category of a sound - necessary for perceptual and cognitive decision-making, unfolds over a longer time window than visual object identification. For example, correct identification response latencies for environmental sounds have been reported to range from 1264 to 3476 ms, with a mean of 2222 ms^9^. Similarly, auditory identification requires additional processing relative to simple detection (i.e., noticing whether a sound occurred), with identification of non-speech sounds taking approximately 420–475 ms longer than simple detection^18^. Accordingly, we selected 1.5 s as a brief but meaningful presentation duration that allows the auditory source to unfold sufficiently for participants to form a perceptual decision, while remaining short enough for efficient use in trial-based behavioral, EEG, MEG, and fMRI paradigms. The recognizability ratings observed in the current norming study indicate that most stimuli were identifiable at this duration.

To facilitate balanced stimulus presentation in subsequent validation tasks, the 291 sounds were initially divided into 12 provisional semantic categories, each containing 21–30 recordings. These labels were organizational placeholders intended solely to ensure diversity of auditory sources during the experiment. Importantly, they did not reflect an assumed semantic or emotional structure. Instead, the final categorical organization of the stimulus set was derived empirically from participant responses collected during the validation experiment.

### Stimuli processing

All sounds were edited to a uniform 1.5-second duration, with fade-in and fade-out effects applied to minimize variability in onset. The audio files were then converted to a mono (i.e., centered in perceived location, to eliminate spatial localization cues), compressed to ensure the loudness of the audios stays consistent over the 1.5 seconds (i.e., bringing the quietest and loudest parts of the audio closer to each other), and normalized to −10 dB relative to full scale (i.e., bringing the average perceived loudness of the audio to −10 dB relative to full scale). Finally, any sounds exhibiting clipping or unnatural alterations were excluded.

### Stimuli norming experiment

#### Participants

A total of 445 participants were recruited via Prolific. Eighty-four did not complete the task or failed attention checks, resulting in a final sample of 361 participants (mean age = 29.67 ± 5.96; 113 females, 237 males, 11 others). Eligibility criteria included: age between 18 and 40 years, native or fluent English proficiency, normal hearing, no color blindness, and no self-reported mental or neurological problems. Only individuals using headphones were permitted to participate.

Participants were randomly assigned to one of three groups, each rating approximately one-third of the dataset. All participants provided electronic informed consent. The study was approved by the Ethics Committee of the University of Muenster (2024-40-AZ) and conducted in accordance with the Declaration of Helsinki. Compensation was £10.5/hour.

#### Behavioral standardization task

The task was implemented using Psychtoolbox^19,20^. Audio clips were presented consecutively, and participants were instructed to rate three dimensions of each clip: startlingness, emotional valence, and recognizability. For each rating, participants used a mouse-operated continuous slider bar with pictorial anchors at either end to illustrate the scale extremes (e.g., a confused face with a question mark for the low end of the recognizability scale). The format was adapted from the Self-Assessment Manikin (SAM), selected for its established reliability and low cognitive demand^21^.

At the beginning of the task, participants were provided with the following instructions. For startlingness: “Please rate how much the audio startled you by moving the mouse to the left if the sound was not startling or to the right if the sound was startling. The amount of change in the position of the toggle on the screen should reflect your degree of confidence in the response.” For emotional valence: “Please rate how the audio made you feel by moving the mouse to the left if you felt negative after hearing it (e.g. disgusted, sad, or angry) and to the right if you felt positive after you heard the sound (e.g. curious or happy). The bigger movement corresponds to stronger emotions.” For recognizability: “Assess how easily recognizable the sound was to you by moving the mouse to the left if the sound was unrecognizable and to the right if the sound was easily recognizable (e.g., keys jingle). The amount of change in the position of the toggle should reflect the degree of your confidence in the response.”

Following the ratings, participants were instructed to type one or two words describing the presented sound. The on-screen instructions read: “Please use the keyboard to type one word or two to describe what you think the audio was. Please try to type this as quickly as possible and limit your input to one or two words at most. Please try to keep your guess as general as possible. For example, if the sound is of tree leaves rustling, please write plants and not tree leaves.” This open-ended format was chosen to capture naturally occurring semantic categories rather than constrain responses to predefined options.

The experimental sequence began with a fixation cross, which was displayed for 600 ms, jittered by 50 ms, followed by a sound (1,500 ms), then a second fixation cross (600 ms, jittered by 50 ms). After the second fixation cross, the three rating scales were shown in a fixed order (startlingness, emotional valence, and recognizability). For each scale, participants had 6 s to respond. Finally, the open-ended question was presented, with a maximum of 8 s to respond. Sounds were presented in a pseudorandomized order such that each author-defined category was represented at least once every 12 trials.

Interleaved between the main trials, 10 attention trials were presented throughout the experiment. The attention trials were pseudorandomized such that at least one occurred within each block of 10 sound trials. In the attention trials, participants were required to correctly select the color of a square presented in the middle of the screen from three options. Participants were required to answer correctly on at least 70% of attention trials; otherwise, their data were excluded. Participants had 60 s to answer the attention question; failure to respond within the time led to immediate termination of the experiment and exclusion from the study.

A rest period was included after 50 standard (sound) trials, corresponding to the midpoint of the experiment. Participants were instructed to take a 5-minute rest if they wished. During this rest phase, a timer appeared on the screen to inform participants of the remaining time before the experiment automatically resumed. Participants were able to terminate the rest by pressing the space bar at any time during this 5-minute period.

A practice block was administered at the start to familiarize participants with the task. At the beginning of the practice phase, an audio clip was played, and participants were asked to adjust the volume until it reached a comfortable level to avoid further adjustments during the experiment. Since all the sounds were standardized for loudness, a single volume adjustment at the beginning was sufficient. After that, the example audio was played again, and participants were asked to rate the audio on the three dimensions and type a one- or two-word description. The example audio used during the practice phase was unrelated to any experimental stimuli. After this practice trial, the main task began.

## Data Records

The data has been deposited on the Open Science Framework^22^. The data are organized in the following folders, files, and archives:

- Meta_Data: This folder contains the Meta_Data.xlsx worksheet, which lists the source of each audio file and its description from the original audio source.
- Processing_results: This folder contains the Audio_Categorization.xlsx and Ratings_Norm.xlsx worksheets.
  - Audio_Categorization.xlsx: This file contains the results of the two-step k-means algorithm that was run to empirically categorize the sounds using the data given by the participants
  - Ratings_Norm.xlsx: This file contains the normalized average ratings of each of the participants for each of the sounds

- Raw_Data: This folder contains the Raw_Data_Ratings.xlsx and Raw_Data_Text.xlsx worksheets, which contain ratings and participants’ text guesses from the standardization study. Each participant was assigned an arbitrary identifier between 1 and 361.
- Sound-Standardization.zip: This folder contains a documented version of the analysis script, preprocessed data, and analysis results. An overview of the code is available in the README.md file.
- Sounds_Freesound.zip: This archive contains the stimuli files obtained from Freesound.org.
- Sounds_BBC: This folder contains source annotations and reconstruction scripts for stimuli derived from BBC Sound Effects. The dataset does not redistribute BBC Sound Effects audio material. The BBC_Sounds.xlsx file contains the URLs of the original BBC source files and the timestamps used to extract the corresponding sound segments. The Pipeline.txt file documents the sound-processing steps used to reconstruct the audio clips used in the validation study. The BBC_Sounds_Pipeline.py script automates the reconstruction process, and the README.md file provides instructions for using the script. Together, these annotations and scripts allow users to identify and reconstruct the corresponding stimuli from the original BBC Sound Effects files. Users are responsible for ensuring that their use of the BBC material complies with the BBC Sound Effects/RemArc Licence.

## Technical Validation

### Rating data quality

Participants who completed fewer than 80% of rating scales were excluded from rating analyses (33 exclusions). For each sound, mean startlingness, emotional valence, and recognizability ratings were computed and z-normalized.

**Fig. 1:**
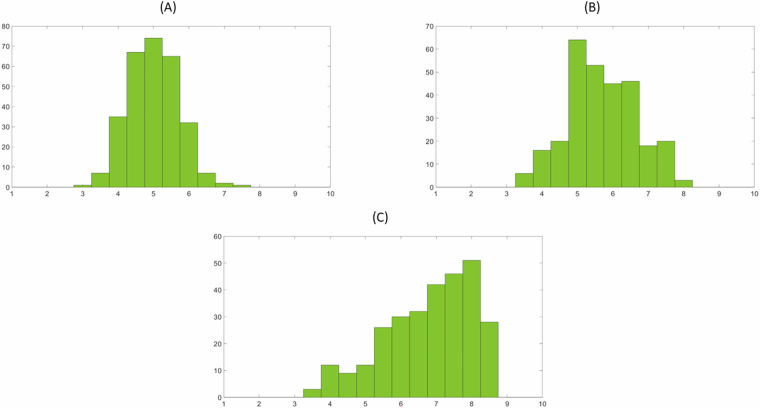
Histogram of the average rating of the sounds’ (**A**) startlingness, (**B**) emotional valence, and (**C**) recognizability.

Normality was assessed using the Shapiro–Wilk test with Lilliefors correction. Startlingness ratings were normally distributed (mean = 5.00 ± 0.70, *D*(291) = 0.03, p = 0.50), whereas recognizability (mean = 6.75 ± 1.22, *D*(291) = 0.09, p < 0.001) and emotional valence (mean = 5.70 ± 0.95, *D*(291) = 0.06, p = 0.03) were not. Both non-normal scales exhibited positive skew: recognizability values indicated high participant confidence in identifying sounds, and emotional valence suggested slightly more positive affective responses overall. Figure 1 displays distributions of the three rating dimensions.

Because recognizability ratings were skewed toward the upper end of the scale, we further quantified the distribution using scale-based thresholds. The midpoint of the recognizability scale was 5; therefore, we calculated the proportion of sounds with mean recognizability ratings above this midpoint, as well as the proportion of sounds exceeding higher thresholds of 6, 7, and 8. These threshold analyses were intended to characterize how many stimuli were rated as generally recognizable and how many approached the upper end of the scale. These analyses indicated that most sounds were rated as highly recognizable, with 73% of sounds exceeding a mean recognizability rating of 6, 51% exceeding 7, and 17% exceeding 8. Only 10% of sounds fell below the midpoint of 5. This distribution suggests that the dataset is particularly well suited for studies requiring clearly recognizable auditory objects. At the same time, the skew toward high recognizability indicates a potential ceiling effect, meaning that recognizability may have limited utility as a continuous differentiating variable across the full stimulus set. The current dataset is therefore better suited for experiments that require recognizable stimuli.

To test the inter-rater reliability a two-way random, average score ICC, which measures inter-rater consistency was calculated. *ICC(2, k)* values were .86, .97, and .95 for startlingness, recognizability, and emotional valence respectively. These results suggest that the mean ratings for each sound provide a very stable and reliable measure of the intended psychological dimensions.

### Text processing pipeline

Free-text responses were cleaned by removing non-sensical entries and stop-words using MATLAB’s built-in list, supplemented with: voice, noise, sound, noises, sounds, voices, and answer. Typos in the text responses were corrected using MATLAB’s native autocorrection tools (“correctSpelling” function). The MATLAB script including the correction procedure is included in the Code Availability section.

We used the participants’ guesses to categorize the sounds. To achieve this, we implemented a two-step procedure. In the first step, we applied semantic embedding^23^, a technique commonly employed in natural language processing (NLP)^24,25^. In this approach, each word is mapped to a multidimensional space to quantify its relationship to all other words in the language’s lexicon. To understand this step, one might consider mapping the words “queen” and “king” into a two-dimensional space. Although these two words are close on one dimension (related to “royalty”), they are different in another dimension (e.g., in “gender”). Using this quantification, one can then categorize the words. Notably, any lexicon will have more than two dimensions, and these dimensions are not as easily interpretable as in our example, since they lack a predefined semantic meaning.

The guesses in our study were transformed into a 200-dimensional GloVe semantic embedding space, trained on ~27 billion words from Twitter^23^. After cleaning the participant data, each answer was entered into the first-step analysis as a separate guess, regardless of the sound associated with it. Each of the 291 sounds received around 126 guesses in total. In the first step, we only clustered unique guesses to maintain a balanced category density and variance and thus preventing the formation of clusters that contain only very few sounds with very similar guesses across most participants. In essence, having the same guesses for some sounds would force the k-means algorithm to find categories with very little variance which would eventually lead the algorithm to categories each sound on its own.

After mapping the guesses into the embedding space, a k-means algorithm was used to categorize them. To determine the optimal number of clusters for the first-step k-means algorithm, the cluster number with the maximum silhouette score was selected. The silhouette score is a standard measure in unsupervised machine learning for selecting the optimal number of clusters and avoiding over- and underfitting^26–28^.

Afterward, for each sound, the number of responses assigned to each cluster was counted. Notably, we used the complete set of guesses rather than just the unique ones to classify sounds based on the full distribution of participant responses. Therefore, repeated guesses were retained to weight clustering outcomes, rather than reducing the analysis to unique guesses. A sound was classified into a cluster if the plurality of responses for that sound fell into that cluster. The k-means algorithm is subject to stochasticity due to the noise in the data. To decrease the impact of noise on the final classification, we conducted clustering 1,000 times using different random seeds. For each run, the optimal number of clusters was identified using the maximum silhouette score and then applied in the k-means algorithm with the same random seed to categorize participants’ guesses.

Finally, we created a representation similarity matrix (RSM) for the sounds based on the clustering results of the guesses for each sound. To do so, a 291 × 291 RSM was constructed; each entry in this matrix represented the normalized number of times two sounds were categorized in the same cluster (i.e., if $a{ij}=10/1000$, it means that sounds *i* and *j* were bundled in the same cluster 10 of 1000 runs). This step closely resembles ensemble learning in supervised learning^29^.

In the second step, another k-means algorithm was applied to the RSM to cluster the sounds. Again, silhouette scores determined the optimal number of clusters. Notably, the second k-means clustering was applied only once, as the first-step output yielded a single RSM with an acceptable signal-to-noise ratio.

Finally, for visualization, an agglomerative hierarchical clustering algorithm was run on the combined RSM. The resulting dendrogram illustrates the relationships between individual sounds within categories and between categories overall.

### Text processing results

**Fig. 2:**
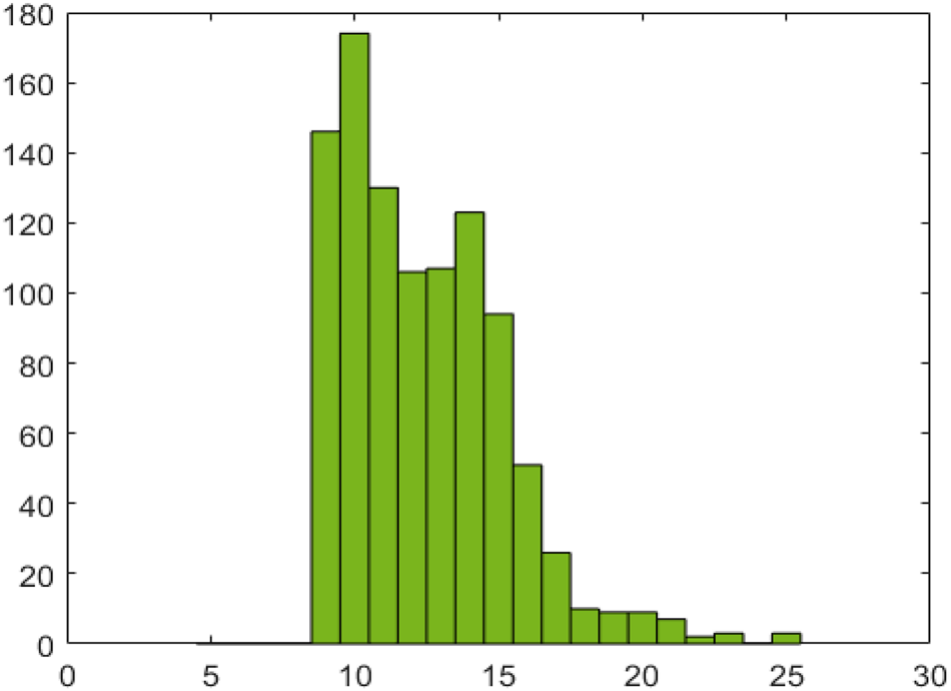
Histogram of the optimal number of categories based on the maximum silhouette score in the first step.

For the first step of sound classification, silhouette scores were calculated to determine the optimal number of clusters for the first-step k-means algorithm. Silhouette scores were computed for 5 to 25 clusters. Figure 2 shows the histogram of the chosen optimal number of categories based on the maximum silhouette score in each run. The histogram shows a clear tendency for the algorithm to cluster the responses into 10 categories.

**Fig. 3:**
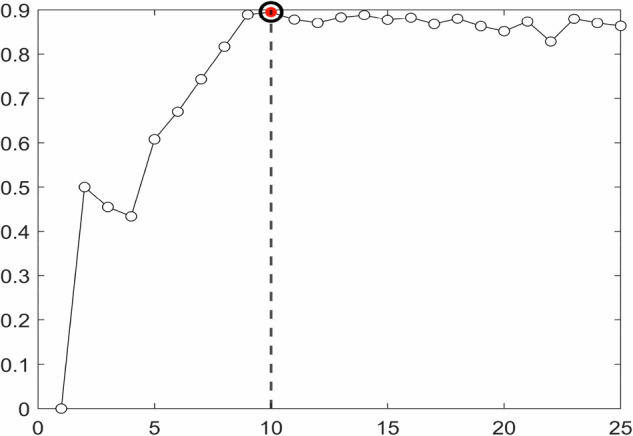
Average silhouette score for the second k-means step clustering sounds based on the combined RSM.

In the second step, the k-means algorithm classified sounds into 10 clusters. The 10 clusters were chosen based on the maximum silhouette score computed across cluster solutions ranging from 1 to 25 (Fig. 3). The average silhouette score for the 10 clusters was 0.89, indicating high reliability of the k-means solution^30^.

Table 1 summarizes the main results of the analysis, showing how each sound was classified into its respective category. The sound labels (e.g., ME01: Mechanical & Electronic sound 1) reflect the original grouping defined by the authors (see the Data Records section for a full list of the author-defined categories). Each row in the table instead represents how the sounds were classified based on the participants’ responses. As shown, most sounds clustered as anticipated by the authors, albeit with a few exceptions, such as the speech category merging with bodily sounds (I.e., SP and BO categories).

**Table 1:** Categorizations of Sounds.

Category	Sound IDs
Category 1	BI10, BI11, BI16, BI13, BI38, MA01, MA03, MA06, MA07, MA08, MA09, MA11, MA12, MA14, MA15, MA16, MA17, MA18, MA19, MA20, MA21, MA23, MA24, MA26, MA27, MA28, MA29, MA30, MA31, OA24, OA25, OA26, SP26, SP33
Category 2	CG43, CG44, CG45, ME10, SO01, SO05, SO07, SO08, SO09, SO10, SO11, SO12, SO13, SO16, SO18, SO21, SO22, SO23, SO25, SO27, SO29, SP01, SP02, SP06, SP22
Category 3	BO01, BO02, BO03, BO04, BO05, BO07, BO08, BO09, BO10, BO11, BO15, BO16, BO17, BO18, BO19, BO22, BO24, BO25, BO26, BO27, BO28, BO29, SO24, SP03, SP05, SP07, SP08, SP11, SP13, SP14, SP15, SP16, SP20, SP21, SP23, SP31, SP34, SP35
Category 4	BS21, BS23, BS24, ME14, ME19, ME25, ME34, ME36, NW16, OA22, SO26, TR01, TR02, TR03, TR04, TR05, TR06, TR07, TR11, TR13, TR14, TR15, TR16, TR17, TR18, TR19, TR20, TR24, TR25, TR26, TR27, TR28, TR29, TR30, TR31, TR32, TR33, TR34, TR36, TR37
Category 5	BS26, MD01, MD12, MD13, MD14, MD17, MD18, MD02, MD20, MD21, MD22, MD23, MD28, MD30, MD31, MD32, MD33, MD35, MD37, MD39, MD04, MD40, MD05, MD08, MD09, SP19, SP32
Category 6	NW13, NW15, NW18, NW26, NW27, NW30, NW31, NW32, NW34, NW35, NW40
Category 7	BI12, BI14, BI15, BI18, BI02, BI20, BI24, BI25, BI27, BI29, BI03, BI30, BI32, BI33, BI34, BI36, BI39, BI05, BI08, OA01, OA02, OA03, OA04, OA05, OA06, OA08, OA09, OA11, OA12, OA13, OA14, OA15, OA16, OA17, OA18, OA19, OA20, OA21, OA23
Category 8	BS01, BS02, BS03, BS04, BS05, BS06, BS07, BS10, BS11, BS12, BS13, BS14, BS15, BS16, BS17, BS18, BS19, BS20, BS22, BS25, ME01, ME02, ME21, ME22, ME23
Category 9	CG01, CG02, CG14, CG33, CG35, NW01, NW02, NW03, NW04, NW05, NW06, NW07, NW08, NW09, NW10, SO17, SO28, SO30
Category 10	BO30, CG16, CG17, CG31, CG32, CG36, CG37, CG38, CG39, CG40, CG41, CG42, CG46, CG48, CG49, CG50, CG51, ME03, ME04, ME05, ME06, ME07, ME08, ME09, ME12, ME20, ME26, ME32, ME33, ME35, NW36, NW39, SO04, SO06

*Note*. Name of the sounds represent the original author-defined categories as follows: ME: Mechanical & Electronic; CG: Household Items; BO: Bodily Sounds; NW: Natural & Water Sounds; BS: Bells & Sirens; MD: Music & Drums; SO: Sports Sounds; SP: Speech Sounds; TR: Transportations; MA: Mammals; BI: Birds; OA: Other Animals.

**Fig. 4:**
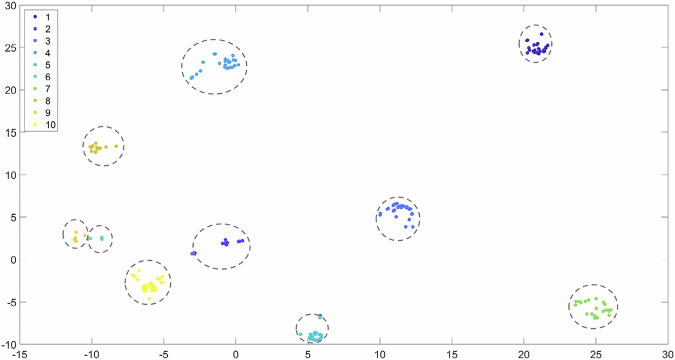
Scatter plot of the sounds based on t-SNE projections. Numbers in the legend correspond to the category numbers in Table 1.

Furthermore, Fig. 4 displays a scatter plot of the sounds (i.e., the combined similarity matrix) and their categorization from the second step, after reducing the matrix to two dimensions using the t-SNE algorithm. This is a non-linear dimensionality reduction algorithm that prioritizes preserving local neighborhoods over global geometry, allowing clearer visualization of clusters^31^.

**Fig. 5:**
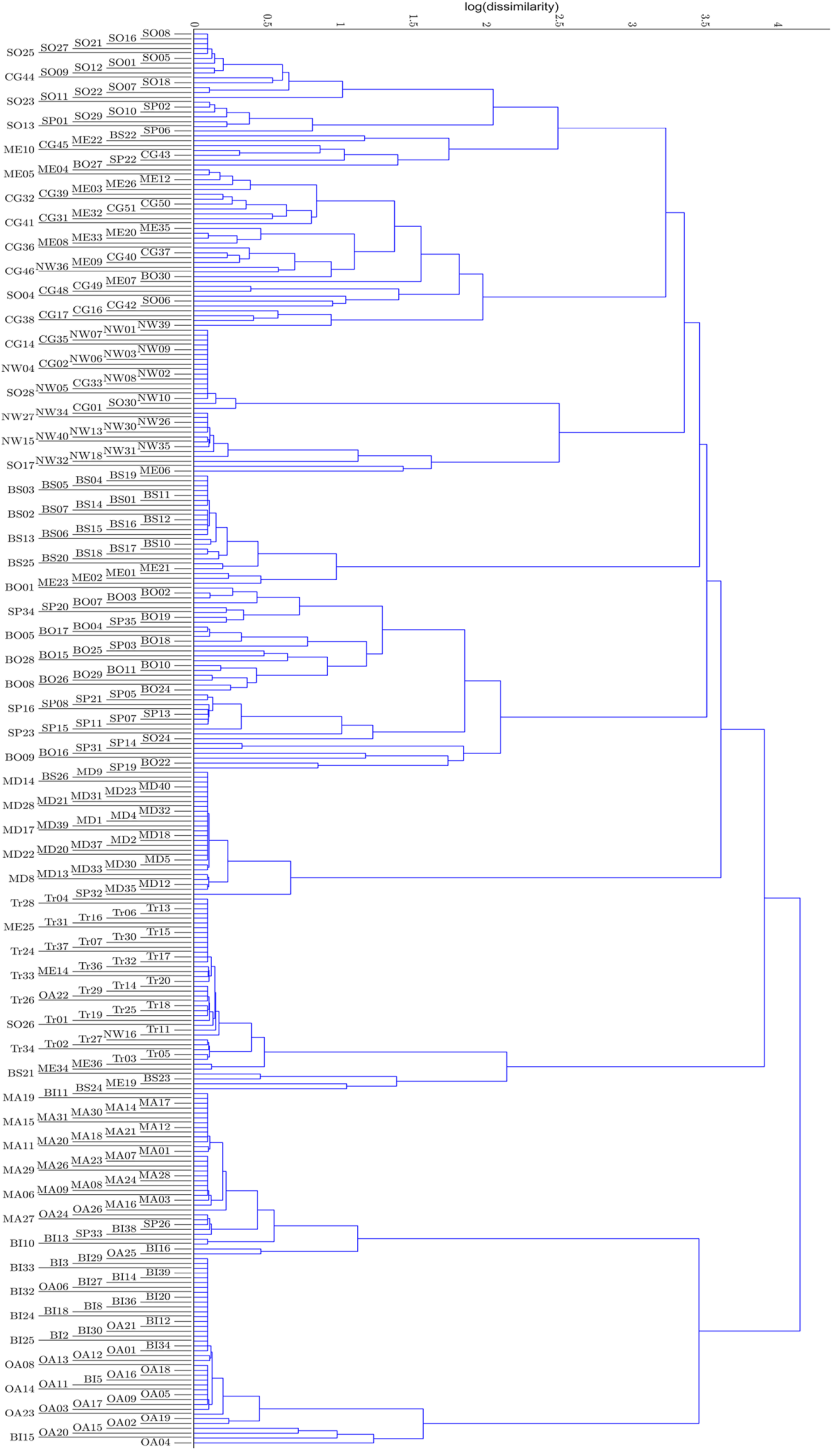
Full Dendrogram of the Sounds.

Lastly, to understand the full relationship between sounds and their categories, Fig. 5 shows a complete dendrogram from hierarchical clustering applied to the combined RSM.

## Usage Notes

Researchers can use the audio dataset based on their research question. ‘Audios_Rating.xlsx’ contains the average ratings of the participants for each of the audio. Audio_Categorization.xlsx contains the labels assigned to each audio. Alternatively, researchers could use the raw rating and text data provided to run their own analyses or train their own machine learning models.

The dataset can be used both to select relatively neutral auditory stimuli and to select stimuli that vary more broadly in affective response. Researchers interested in neutral stimuli may select sounds with valence ratings close to the scale midpoint and low or moderate startlingness ratings. Conversely, researchers interested in affective variability may select sounds spanning a broader range of valence and startlingness values.

The startlingness ratings should be interpreted as perceived startlingness or startle-like response, rather than as a canonical measure of arousal. This distinction is important because startlingness refers more specifically to the extent to which a sound is perceived as sudden, salient, or capable of eliciting an immediate orienting or defensive response. This dimension is particularly relevant for cognitive neuroscience and neuroimaging research, where sudden or startling sounds may influence early attentional orienting, motor preparation, physiological responses, and neural activity^32–34^. Thus, the startlingness ratings provide a useful stimulus-control dimension for researchers who wish to avoid, match, or intentionally manipulate immediate startle-like responses across auditory conditions.

For the valence score, participants were asked to rate how they felt about the sound based on a bipolar negative to positive scale by moving the toggle left or right. The participants were instructed to use the distance from the midpoint as an indicator of the intensity of their experienced emotion. Thus, although the sign of the rating values reflects the polarity of the emotions induced by the presented stimuli, the distance from the scale midpoint can be interpreted as an index of emotional intensity, regardless of whether the emotions are positive or negative. Therefore, researchers interested in emotionality may derive this measure by using absolute emotional valence ratings, i.e., the absolute distance between each sound’s rating and the neutral midpoint.

## Data Availability

The data and the code used for the analysis are available from the Open Science Framework^22^.

## Code availability

The code for the project can also be found on the following GitHub link: https://github.com/ajn3333/Sound-Standardization.
